# Extracellular mutation induces an allosteric effect across the membrane and hampers the activity of MRP1 (ABCC1)

**DOI:** 10.1038/s41598-021-91461-3

**Published:** 2021-06-08

**Authors:** Yuval Bin Kanner, Assaf Ganoth, Yossi Tsfadia

**Affiliations:** 1 The School of Neurobiology, Biochemistry and Biophysics, George S. Wise Faculty of Life Sciences, Tel Aviv University, 69978 Tel Aviv, Israel; 2 The Interdisciplinary Center (IDC), P.O. Box 167, 4610101 Herzliya, Israel; 3 Department of Physical Therapy, School of Health Professions, Sackler Faculty of Medicine, Tel Aviv University, 69978 Tel Aviv, Israel

**Keywords:** Molecular modelling, Computational biophysics, Membrane proteins

## Abstract

Dynamic conformational changes play a major role in the function of proteins, including the ATP-Binding Cassette (ABC) transporters. Multidrug Resistance Protein 1 (MRP1) is an ABC exporter that protects cells from toxic molecules. Overexpression of MRP1 has been shown to confer Multidrug Resistance (MDR), a phenomenon in which cancer cells are capable to defend themselves against a broad variety of drugs. In this study, we used varied computational techniques to explore the unique F583A mutation that is known to essentially lock the transporter in a low-affinity solute binding state. We demonstrate how macro-scale conformational changes affect MRP1’s stability and dynamics, and how these changes correspond to micro-scale structural perturbations in helices 10–11 and the nucleotide-binding domains (NBDs) of the protein in regions known to be crucial for its ATPase activity. We demonstrate how a single substitution of an outward-facing aromatic amino acid causes a long-range allosteric effect that propagates across the membrane, ranging from the extracellular ECL5 loop to the cytoplasmic NBD2 over a distance of nearly 75 Å, leaving the protein in a non-functional state, and provide the putative allosteric pathway. The identified allosteric structural pathway is not only in agreement with experimental data but enhances our mechanical understanding of MRP1, thereby facilitating the rational design of chemosensitizers toward the success of chemotherapy treatments.

## Introduction

**Figure 1:**
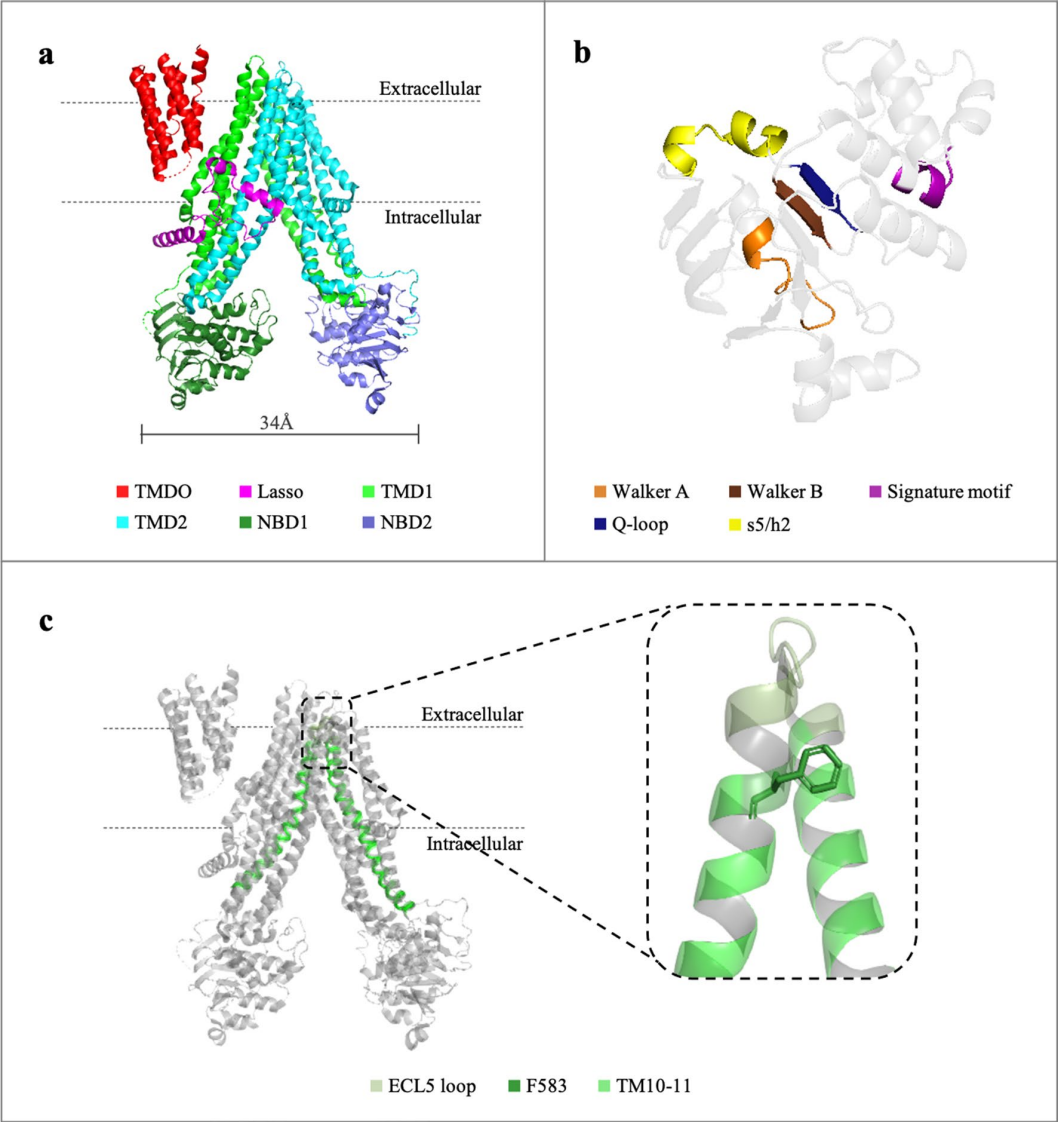
Cartoon representations of the apo inward-facing conformation of MRP1 (PDB: 5uj9). **(a)** Protein’s domains: TMD0, Lasso motif, TMD1, TMD2, NBD1, and NBD2 are colored in red, pink, light green, cyan, dark green, and blue, respectively. **(b)** Known motifs in NBD2 necessary for ATP binding and hydrolysis: Walker A, Walker B, Signature motif, and Q-loop colored in orange, brown, purple, and dark blue, respectively. s5/h2 loop is also mentioned in yellow. **(c)** Helix 10 and helix 11 are highlighted. Inset: The location of F583 in the ECL5 loop between helix 10 and helix 11 of TMD1 is indicated in sticks representation.

Multidrug resistance-associated protein 1 (MRP1/ABCC1) is a member of the superfamily of ATP-binding cassette (ABC) transporters. In healthy tissues, MRP1 fulfills important roles such as efflux of xenobiotics and endogenous metabolites and transport of inflammatory mediators^1^. However, in cancer cells it is overexpressed, resulting in resistance to anti-cancer drugs. It is especially prevalent in neuroblastoma and cancer cells found in the lung, breast, and prostate^2^. The 1,531 amino acids human transporter is composed of three transmembrane domains (TMD0, TMD1, and TMD2), two nuclear binding domains (NBD1 and NBD2), and an interfacial domain known as the Lasso motif^3^ (Fig. 1A). TMD1 and TMD2 are composed of six helices each and together form the transport path. NBD1 and NBD2 are structurally and functionally nonequivalent, with only one competent ATPase site^4^. It has been demonstrated that while both ATPase sites bind ATP, hydrolysis occurs only at NBD2. Each of the ATPase sites consists of Walker A and B motifs from one NBD and the signature motif from the other (Fig. 1B). Walker A and the signature motif are responsible for ATP binding while Walker B has a role in ATP hydrolysis. In addition, the Q-loop is known for mediating TMD-NBD interactions^5,6^. Another noticeable element is the s5/h2 loop that is part of the TMD-NBD2 interface, where a cytoplasmic helix from the TMD settles into a socket on the NBD surface (Fig. 1B)^3^.

In this study we used the inward-facing state of the apo-protein, in which the protein is open to the cytoplasm, waiting for ligand recruitment (PDB: 5uj9), for investigating how a single mutation, F583A, affects the dynamics of the protein. In the initial resting state, the NBDs are widely separated, and the translocation pathway is continuous with the cytoplasm, ready for ligand binding^3^. As previously described in the cryo-EM studies, MRP1 shifts between multiple inward-facing conformations in the absence of ligand^7^. Ligand binding induces movement of the TMDs towards each other, to form a high-affinity ligand-binding site^3^. The TMDs movement brings the NBDs closer and aligns them for proper future dimerization. NBD1 binds one ATP molecule and then NBD2 binds the second one, forming an NBD-dimer. The dimerization initiates a further conformational change of the protein to an outward-facing state. Consequently, the affinity of the ligand reduces, and it is released to the extracellular side of the membrane. In the final step of this process, ATP hydrolysis occurs in NBD2 followed by dissociation of ATP (from NBD1) and ADP and Pi (from NBD2) and the protein returns to its initial inward-facing state^4^.

While the schematic formalism of the translocation is clear, the details of the reaction mechanism are yet to be elucidated. Our previous experimental data showed that the point mutation F583A essentially eliminates organic anion transport and impairs the release of ADP, thus effectively locks the transporter in a low-affinity binding state^8^. F583 is located in the extracellular side of the protein and is at the C-terminus end of ECL5 as it turns into helix 11 (Fig. 1C). It is the first outward-facing amino acid in MRP1 found to be critical for the protein’s transport. The F583A mutation disrupts MRP1’s function through a long-range allosteric effect from the ECL5 loop to the cytoplasmic NBDs^8^. It is still unclear how the mutation affects the intracellular NBDs by propagating across the membrane.

Allostery, a well-known notion in biology, largely involves conformational and functional alterations in individual proteins. Because of the paramount role allostery takes place in the function and dynamics of proteins, it is not surprising it is associated with pathologies and diseases. Although an allosteric effect is mostly associated with effector molecules, it can also be impaired through mutation, in the regulatory site, and even in residues spread along the protein. Several thermodynamics methodologies were used to identify key residues in protein allosteric transition^9,10^. One of the most used methods to study the allosteric communication pathways is molecular dynamics simulations, e.g.,^11^. Examining the dynamics of the system enables mapping the potential allosteric site and its communication with the functional site^12^.

Herein, we tackled previously unanswered fundamental questions regarding the protein’s mechanism. We explain, on an atomic resolution scale, how a single mutation can substantially decrease the transport activity of MRP1. We suggest that the exofacial F583A mutation induces a long-range allosteric effect (over 75 Å) ranging from the ECL5 loop to the cytoplasmic NBD2, through helix 10. The mutation unravels the secondary structure of the TMD/NBD2 interface, thus disrupting the signaling between the two functional parts of the protein. Moreover, we show unraveling of the well-known Walker A, Walker B, and Q-loop elements, that were previously shown to be critical for ATP binding and hydrolysis as well as in the s5/h2 loop element. As a consequence, the appropriate conditions for ADP release after ATP hydrolysis may be disrupted and the protein will be stuck in an inactive state. Our findings exemplify how an allosteric effect affects the protein dynamics, consequently hampering its translocation activity.

## Results

### Homology modeling

Homology modeling of membrane proteins, such as MRP1, is similar to that of water-soluble proteins, and modeling membrane proteins results are as adequate as those obtained when modeling their water-soluble counterparts, for example^13,14^. Given that the human and bovine MRP1 share a high sequence identity (91%) and similarity (99%) (E-value lower than approximately 5∙10^–324^), the latter was selected as a template for the homology modeling process. This sequence resemblance between the proteins ensures a high success of the modeling process.

### Model quality assessment

Before the MD simulations, the human MRP1 model structure was evaluated for its quality. The Ramachandran plot showed that 99% of the residues lie in allowed regions, from which 87% in the most favored regions. No major stereochemical clashes or bad contacts for main-chain or side-chain parameters were detected. All bond lengths, backbone, and rotamer angles were in good agreement with standard values. Thus, the resulted model of the homology modeling procedure was a relatively reliable structure.

## Macroscopic view: structural characterization of the protein’s global dynamics

### Overall three-dimensional structure

**Figure 2:**
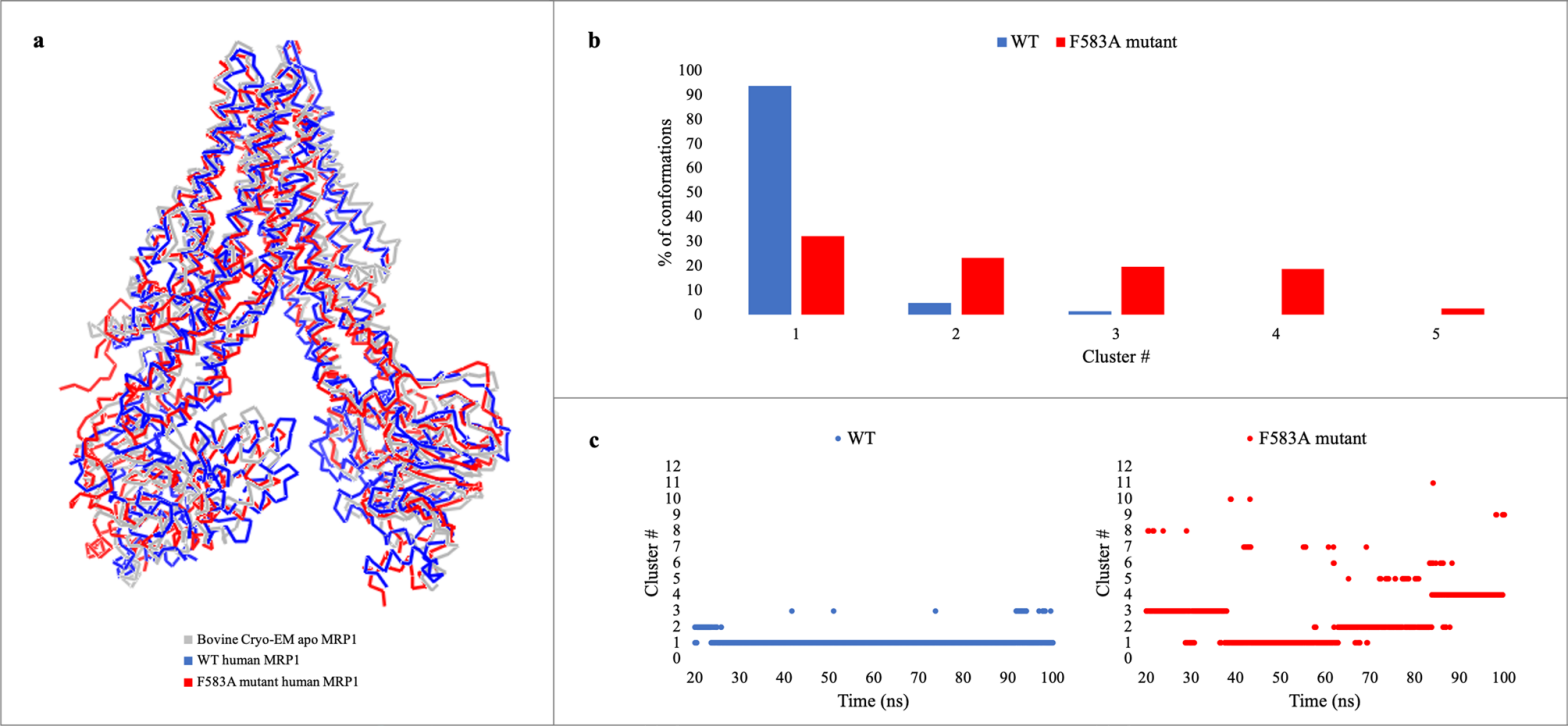
Cluster analysis of the WT (blue) and F583A mutant (red) simulations. **(a)** Superposition of the bovine cryo-EM apo structure (gray), and the representative conformation (cluster #1) of the WT and F583A mutant. **(b)** The percentage of conformations (from all the conformations) in each cluster was calculated. **(c)** Cluster number in a relation to the simulation time, in representative WT and F583A mutant trajectories.

A series of MD simulations up to 0.6 µs of the human WT and F583A mutant in a hydrated lipid bilayer was performed. To ensure the selection of a representative structure, a cluster analysis was performed to select a conformation for further analyses. Representative structure from the WT and F583A mutant, superimposed on to bovine MRP1, is presented in Fig. 2A. We found that our proposed model of the human MRP1 displays the typical topological architecture of eukaryotic ABC transporters. It is composed of 12 TM helices distributed among two domains: TMD1 (helices 6–11) and TMD2 (helices 12–17). The 12 helices from TMD1 and TMD2 are arranged into two pseudo-symmetric bundles: bundle 1, comprising helices 6, 7, 8, 11, 15, and 16; and bundle 2, comprising helices 9, 10, 12, 13, 14, and 17. The two helical bundles span the lipid bilayer and are opened to the cytoplasm. The two NBDs, which are located about 30 Å down into the cytoplasm, are completely separated from each other to about 34 Å. Overall, the suggested model highly resembles the bovine MRP1 and other eukaryotic ABC transporters and retains its distinctive arrangement and characteristics.

It is possible to identify the various conformations that the protein assumes during the simulations through cluster analysis of the structures. Accordingly, our 3 Å cut-off cluster analysis results show that the F583A mutant protein acquires more conformations during the simulation, in comparison with the WT protein. 94% of the WT protein’s conformations belong to cluster #1, presenting one main stable conformation. In contrast, only 32% of the mutant protein conformations belong to cluster #1, which implies its instability in relation to the WT (Fig. 2B). The WT protein appears to be more rigid than the F583A mutant that is more flexible and less stable. Also, The WT protein converged spontaneously and relatively quickly into a stable conformation after ~ 30 ns and it maintains that form to the end of the simulation. On the contrary, the mutant protein fluctuates between different conformations and diverges during the entire simulation (Fig. 2C). These findings support that the WT protein is more stable than the mutant.

### Evolutionary conservation

**Figure 3:**
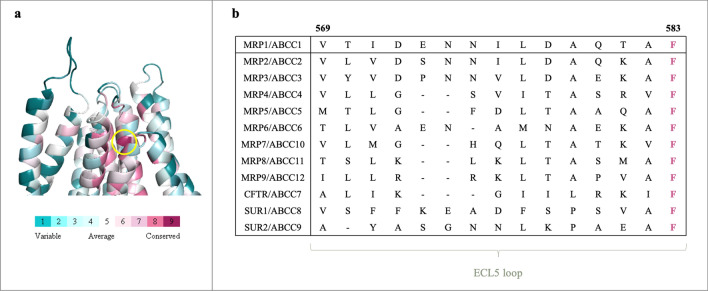
Conservation analysis of F583. **(a)** Cartoon representation of the extracellular part of the protein, colored in cyan to purple scale, from the most variable residue to the most conserved residue, respectively. F583 is colored by dark purple (score: 9), presenting the highest conservation score, and is indicated by a yellow circle. **(b)** Sequence alignment of the ECL5 loop of the human MRP1 and its homologs from the ABCC subfamily, illustrating high conservation of F583 in comparison with the other residues.

We found that the most conserved residue in the extracellular region of the protein is F583 (Fig. 3A). It was rated similarly to highly conserved sequence elements in the protein, such as Walker A, B, and the signature motifs, from both NBDs, that are crucial for the ATPase function of MRP1. Since conservation is highly predictive in identifying catalytic sites and residues near bound ligands^15^, F583’s high conservation score implies its importance for the function of MRP1. Substitution of conserved amino acids was found to lead more frequently to loss of function than substitutions of non-conserved residues^16^.

The level of conservation is based on the evolutionary relatedness between MRP1 and its homologs. The human ABCC subfamily of transporters contains 13 members from the ABC superfamily. The ABCC subfamily includes the Cystic Fibrosis Transmembrane conductance Regulator (CFTR/ABCC7), two Sulfonylurea Receptors SUR1/ABCC9 and SUR2/ABCC9, and nine MRPs^17^. Using ClustalW, sequence alignment of the ECL5 loop (connecting helices 10–11) of the ABCC homologs revealed the invariant conservation of F583, in contrast to other residues in the ECL5 loop (Fig. 3B). Given the high evolutionary conservation of F583 compared to the rest of the ECL5 loop, it is reasonable to assume that F583 may play a major role not only in the protein’s function but in maintaining its structure as well.

### Collective motions of MRP1

**Figure 4:**
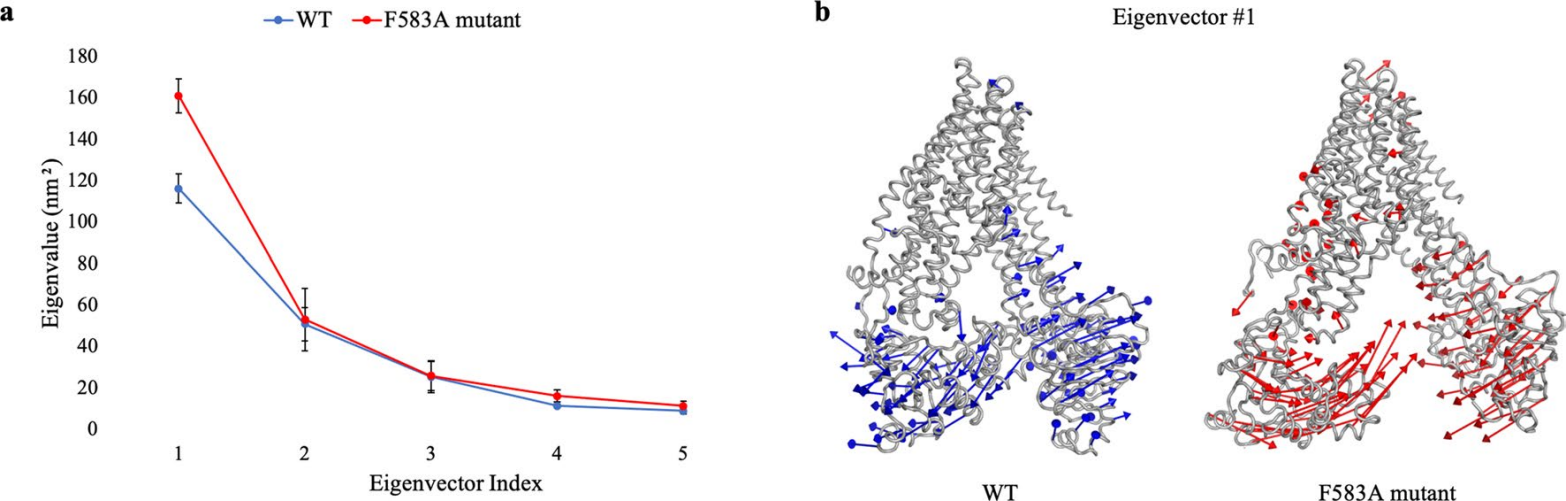
Principal Component Analysis (PCA) of WT (blue) and F583A mutant (red) MRP1. **(a)** The eigenvalues plotted against the first five corresponding eigenvector indices were obtained from the Cα covariance matrix constructed from the MD trajectory. Eigenvalues are sorted on descending order, the first eigenvector presents the largest variance in relation to the other eigenvectors. **(b)** Visualization projection of the obtained results is represented by arrows to show the principal components of the protein’s motion throughout the simulations. The lengths of the arrows are proportional to the size of the movement.

The correlated modes of the protein’s motion during the simulations were calculated by Principal Component Analysis (PCA). This analysis filters global, slow, and probable concerted motions, that contribute most to the total atomic displacement. The first five eigenvectors were considered for this analysis, as they are the most important to describe the significant motions of the protein. The covariance matrix of atomic fluctuations was diagonalized for predicting the eigenvalues. The eigenvalues versus the corresponding eigenvector for WT and F583A mutant proteins are shown in Fig. 4A^18^. During the simulations, the WT and the mutant had similar eigenvalues for eigenvectors two to five. However, the F583A mutant showed higher correlated motions (161 ± 8 nm^2^) in the first eigenvector compared with the WT protein (116 ± 7 nm^2^), as presented in the covariance matrix. The first eigenvalue corresponds to the maximum variance of the projected points. Therefore, a dynamic comparison of the first eigenvector of the WT and the F583A mutant was conducted.

Visualization projection of the first eigenvector, presenting the greatest difference between the WT (blue) and the F583A mutant (red) in the NBDs area is shown in Fig. 4B by a porcupine-like representation. The lengths of the arrows indicate the degree of the significance of the motion; the longer the arrow, the motion is more significant, and vice versa. In the WT protein, the NBDs moving away from each other, creating a putative path for ligand entrance, as expected compared to the transport mechanism (see “Introduction”). By contrast, the NBDs of the F583A mutant are moving in the opposite direction, towards each other, and by that deviate from the transport mechanism and may prevent proper binding of a ligand. NBD1 and NBD2, which are connected by a flexible hinge, move in opposing directions at the WT protein and the F583 mutant. This striking difference between the correlated significant motions of the protein exemplifies how the single mutation F583A induces changes in the dynamic properties of domains that are 34 Å apart from each other, and 75 Å away from the actual location of the mutation.

## Microscopic view: structural characterization of helices 10–11 and NBD2

To account for the F583A mutation, we focused on the structural alterations of helices 10–11 since ECL5 is located between them. Then, we analyzed the conformational changes of NBD2, followed by the interaction between helix 10 and NBD2, because of F583A’s effect on ATPase activity in NBD2.

### Mutual progression of helices 10–11 and the protein

**Figure 5:**
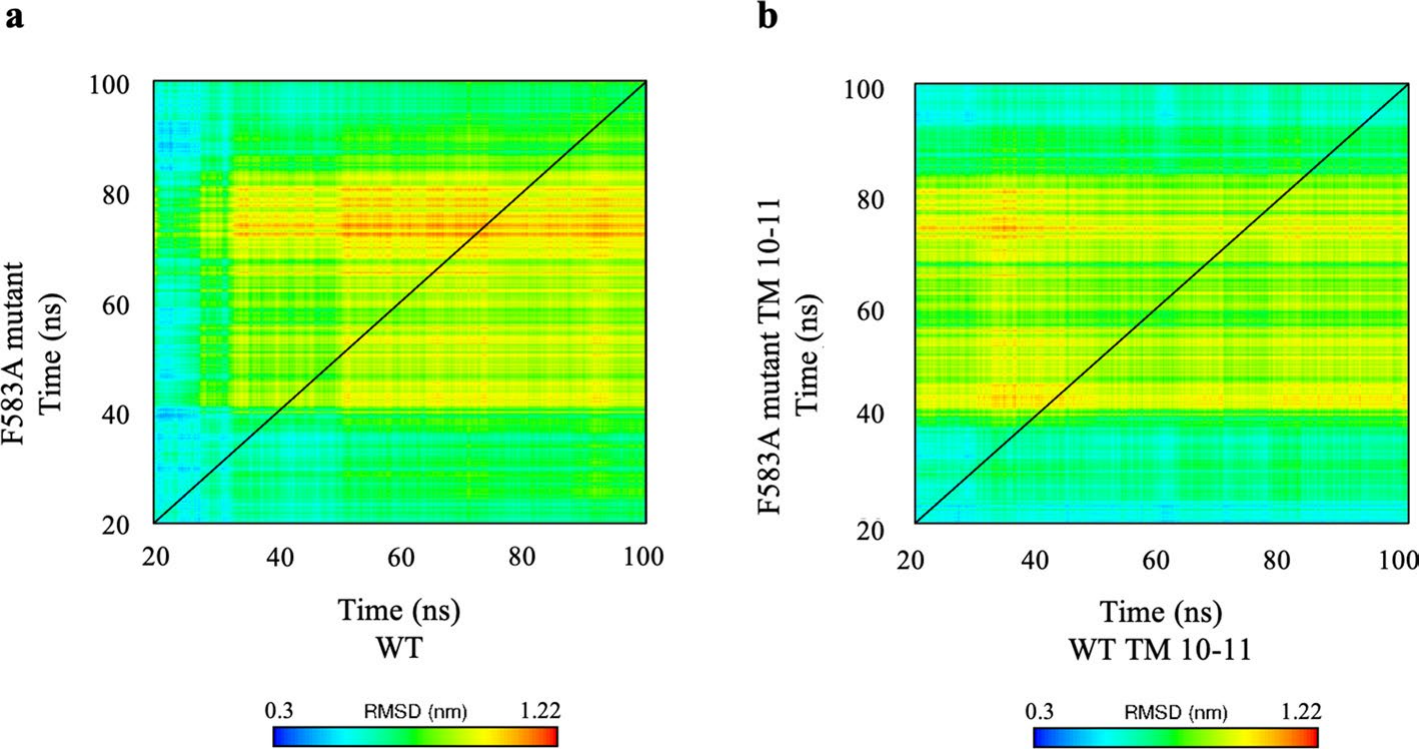
2D-Matrix representation of mutual backbones atoms’ RMSD of the **(a)** WT-MRP1 and F583A-MRP1 mutant and **(b)** WT's helices 10–11 and F583A mutant's helices 10–11, upon a comparison of representative simulations. The RMSD values of the mutant’s trajectory were calculated in relation to those of the WT. The values are given by color codes; blue and red represent high and low similarity, respectively, ranging from 0.3 to 1.22 nm. The diagonal best explains the mutual evolution of the stability of the WT’s and the F583A mutant’s trajectories.

Dynamic comparison of the two-dimensional RMSD (2D-RMSD) of the WT and F583A mutant trajectories is shown in Fig. 5. This two-dimensional RMSD matrix analysis reflects the structural changes of the proteins (Fig. 5A) and of helices 10–11 solely (Fig. 5B) along the simulation time and assesses their stability. The backbone atoms׳ RMSD of the F583A mutant trajectory is presented in relation to the backbone atoms׳ RMSD of the WT protein trajectory. A color code is used for visualizing the RMSD values, colored from blue to red covering the range from 0.3 to 1.22 nm (Fig. 5). The color pattern exhibits progressive yet reversible conformational changes, indicating that both structures are fluctuating and the similarity between them varies.

The mutual evolution of the conformational changes is best manifested when following the diagonal line divided into three phases: a relaxation phase (from ~ 20 to 30 ns and ~ 20 to 40 ns for the WT and the mutant, respectively), a progression phase (~ 30 to 80 ns and ~ 40 to 80 ns for the WT and the mutant, respectively), and a quiescent phase (from ~ 80 ns until the end of the simulations). The relaxation phase is represented by the dark blue, pale blue, and greenish colors stretching from the bottom of the figure. At this phase, the MRP1 protein at both simulations responds to the absence of the packing forces present at the crystal lattice. Both WT and mutant proteins relax at native physiological membrane conditions. At the progression phase, the F583A mutant undergoes conformational changes (shown as ~ 40 to 80 ns at the ordinate), rendering it to be less similar to the evolving WT protein which is relatively stable. Consequently, both conformations become more and more distinct one from the other, as indicated by the yellowish-reddish hues. Finally, at the quiescent phase, the mutant MRP1 continues to evolve, interestingly becoming more resembled the WT protein that keeps stable. The higher degree of similarity between the WT and mutant conformations, as reflected by the smaller RMSD values one with respect to the other, is observed at this phase (represented by the greenish hue).

Notably, as the simulations progress, the WT protein experiences limited changes and is more stable, whereas the mutant MRP1 fluctuates between conformations, as demonstrated by our further analyses. The noticeable similarity between the two matrices, the whole protein (Fig. 5A) and helices 10–11 solely (Fig. 5B), suggests that the progression of the protein is mainly shaped by the progression of helices 10–11. It is of interest to point out that the structures of the protein at both simulations do not co-evolve in parallel simultaneously at the same time and hence the two halves of the matrix are not identical albeit similar.

### A change in the position and orientation of helix 10

**Figure 6:**
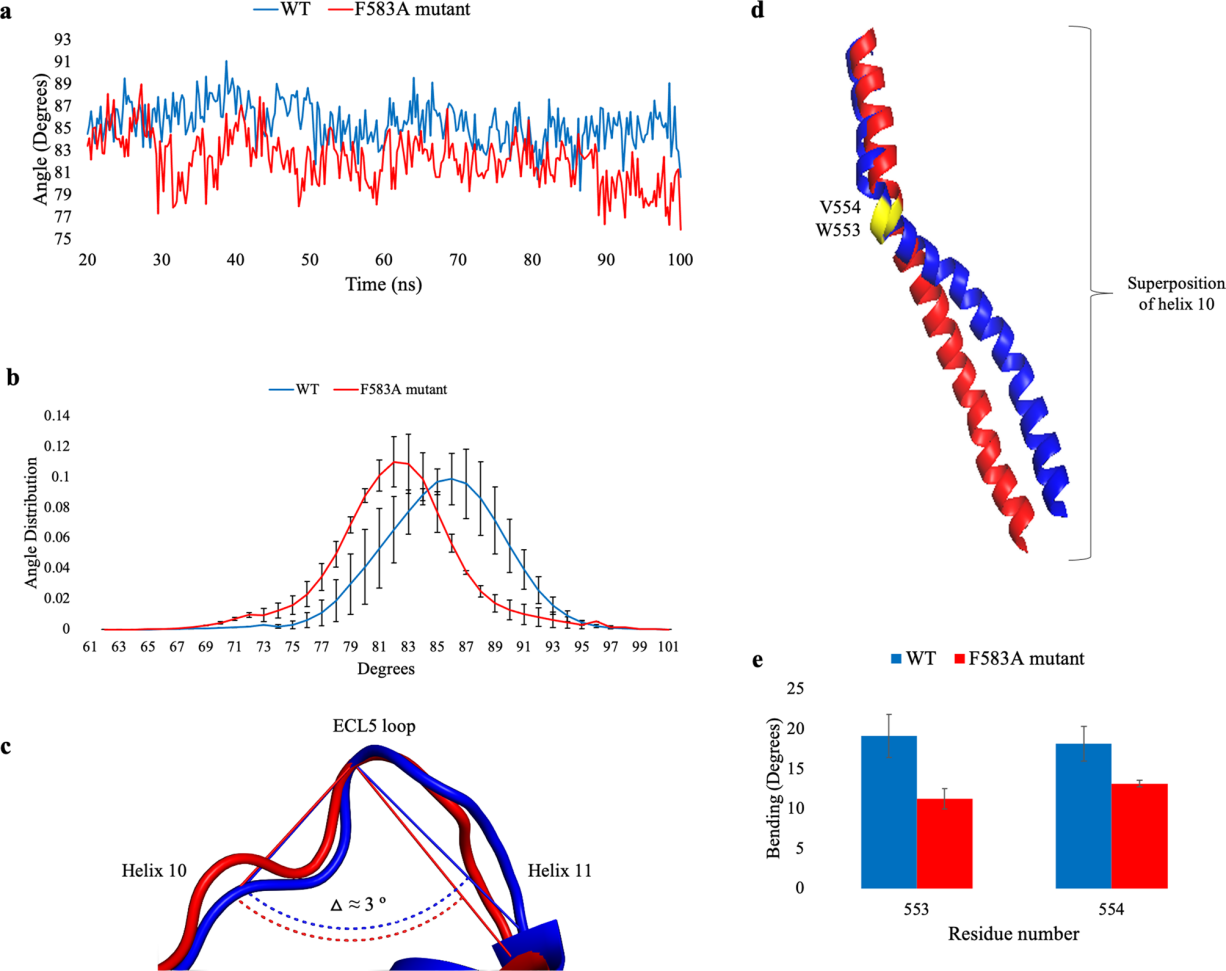
Measurements of the interhelical angle between helices 10–11 (**a–c**) and the bending of helix 10 in the WT (blue) and F583A mutant (red) (**d,e**). **(a)** The interhelical angle between helices 10–11 (degrees) as a function of simulation time (ns) showing a wider dynamic angle in the WT protein in comparison with the mutant. The averages of the three WT and the three F583A mutant simulations are presented. **(b)** Distributions of the average interhelical angle’s values (degrees) of the three WT and three F583A mutants are presented. The F583A mutant’s distribution is narrower and shifts to the left compared to the WT’s distribution, showing smaller angles than in the WT. The averages of the three WT and the three F583A mutant distributions are presented. **(c)** Representative cartoon superposition of the opening angle between helices 10–11 for the WT and the F583A mutant, calculated by defining vectors as the angle axes. The delta (△ ≈ 3 º) represents the difference in the interhelical angle. **(d)** A representative cartoon comparison presentation of the bending of helix 10 in the WT and F583A mutant. Residues W553–V554 are colored in yellow, presenting the starting point of helix 10’s bending. **(e)** Residues W553–V554 degree of bending in the WT and F583A mutant. The averages of the three WT and the three F583A mutant simulations are presented.

A close inspection of how the WT protein's and the F583A mutant's conformations evolve throughout the simulations reveals high similarity; in both cases, the transmembrane α-helices and the angles between are retained and kept, except from the angle between helices 10 and 11 and the angularity of helix 10. Hence, to account for the difference between the WT protein and the F583A mutant we measured the interhelical angle between helices 10 and 11. We found that for the F583A mutant, the average angle (82.2 ± 2 º) is smaller than the average angle of the WT (85.4 ± 2 º), as measured during the trajectories of the three independent simulations (Fig. 6A). Notably, the distribution of the angle's values of the WT is more flattened and is located in close vicinity to the right of the mutant's distribution, meaning the WT is more opened to the cytoplasm than the mutant (Fig. 6B). The two distributions showed significant difference (p-value is < 0.00001). When looking at the most common cluster, one can detect the difference in the degree of openness of the angle between helices 10–11 showing a 5 º difference favoring the WT (Fig. 6C). To reveal the cause for the change in the interhelical angle we performed structural analyses of helices 10 and 11. Since helix 10 is composed of two rod-like elements, connected by W553 and V554, we followed the angularity of the helix. In the WT protein helix 10 bends at residues W553-V554 and provides a more open conformation, in comparison to the F583A mutant’s helix (Fig. 6D). The bending angle in helix 10 is 19 ± 3 º for W553 and 18 ± 2 º for V554, while in the F583A mutant the angles are only 11 ± 1 º and 13 ± 0.4 º, respectively (Fig. 6E). The difference in the bending of helix 10 propagated to the edge of helix 10, changing the docking orientation of the helix on NBD2. Both the changes in the opening angle and the bending of helix 10 support our macroscopic results, showing relatively closure of the transport path of the mutant when compared to the WT.

### Secondary structure motifs in NBD2

**Figure 7:**
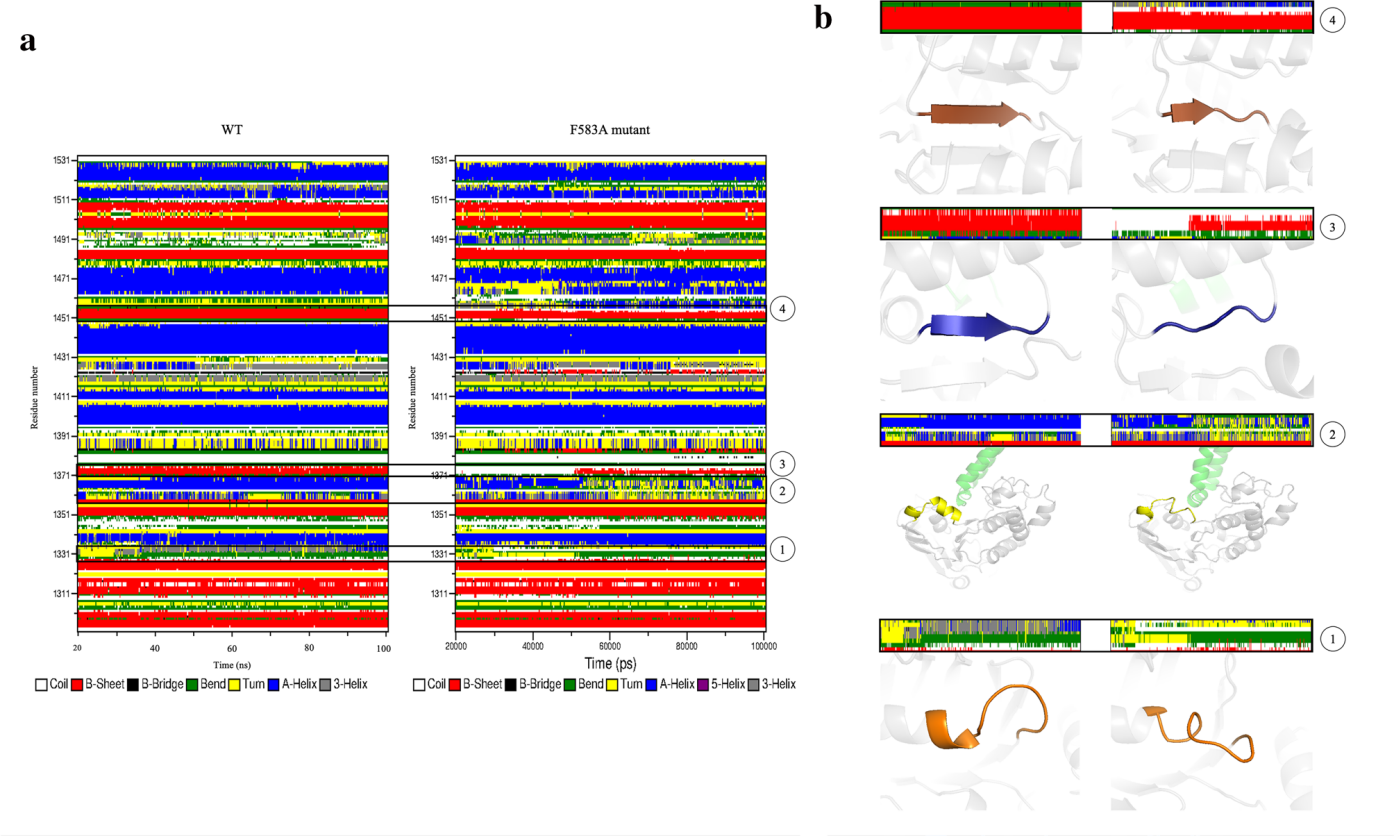
Secondary structure analysis of NBD2 is shown using the STRIDE algorithm. **(a)** DSSP colored map representing the secondary structure elements of each residue as a function of time. Color code representing the structure is shown beneath the graphs. **(b)** A zoom in snapshot of four key elements in NBD2 numbered according to their occurrence from N- to C-terminus: (1) Walker A, (2) s5/h2 loop, (3) Q-loop, and (4) Walker B.

To account for the secondary structure differences in NBD2 between the WT and the F583A mutant DSSP analysis for this domain was performed (Fig. 7A). In general, the main differences between the NBD of the WT and the mutant were in the most common secondary structures, α-helices, β-sheets, and turns. The number of residues forming the α-helices and β-sheets is larger in the WT structure when compared to the F583A mutant. There is a difference of five residues and one residue for the formation of α-helices and β-sheets, respectively. By contrast, three other residues in the mutant take part in the formation of turns, causing changes in the direction of the polypeptide chain.

NBD2 consists of conserved motifs that are essential for nucleotide binding, hydrolysis, and NBD-NBD as well as NBD-TMD communication. Zooming in, secondary structural differences in four of those motifs were detected in the F583A mutant. (1) Walker A—unraveling of a helix in residues K1333-S1334, (2) s5/h2 loop—unraveling of an α-helix in residues L1363-K1369, (3) Q-loop—unraveling of a β-strand in residues I1372-P1374 that are located in the starting point of the loop, (4) Walker B—unraveling of a β-strand in residues L1453-D1454 (Fig. 7B). Walker A is responsible for ATP binding (phosphates binding). The conserved K1333 forms H-bonds with the oxygen atoms of the α- and β-phosphates, thereby holding both phosphates in a defined orientation. S1334 forms a H-bond with a D1454 residue of Walker B and interacts with the magnesium ion and the β-phosphate of the bound ATP^19^. The loss of the α-helix may interrupt the formation of H-bonds with the incoming ATP phosphates. Walker B takes part in ATP hydrolysis. The changes in the secondary structure, the unraveling of the β-strand, deviating from the conserved secondary structure element, may interrupt the hydrolysis mechanism. The F583A mutation also unravels the β-strand element of the Q-loop, that couples TMD-NBD communication^5,6^. Moreover, the most significant change was in the s5/h2 loop which is part of the TMD-NBD2 interface, where the cytoplasmic segment of helix 10 from the TMD1 settles into a socket on the NBD2 surface. It was previously shown that a main secondary structural difference between the degenerate ATPase site (NBD1) to the consensus ATPase site (NBD2) is the lack of s5/h2 element in the degenerated site^3^. Moreover, the absence of s5/h2 in the TMD-NBD1 interface renders it to be weaker than the TMD-NBD2 interface. Here, we show the absence of the α-helix h2 and suggest that similarly to NBD1, the lack of this helix impairs the strength of the interaction between TMD-NBD2. Overall, we demonstrate that the F583A mutation causes major changes in critical elements for proper ATPase activity.

### The interaction between helix 10 and NBD2

**Figure 8:**
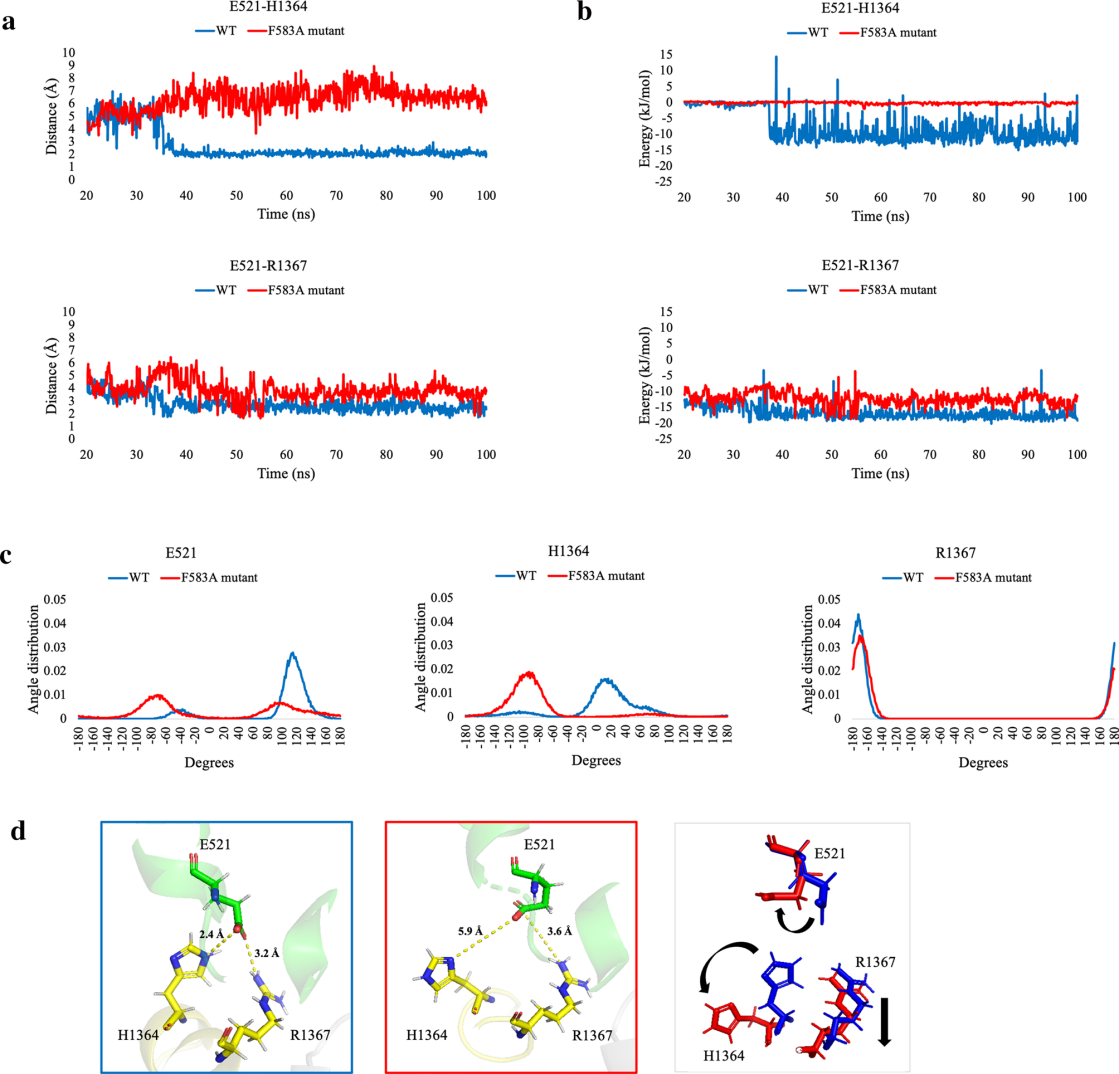
The interaction between helix 10 (E521) and NBD2 through the s5/h2 element (H1364 and R1367). **(a)** Dynamic distance analysis comparison of the distances between E521-H1364 and E521-R1367 between the WT and the F583A mutant. **(b)** Total energy (vdW + Electrostatic) calculations for E521-H1364 and E521-R1367. **(c) **Dihedrals’ analysis comparison of E521, H1364, and R1367 between the WT and F583A mutant. E521 and H1364 showed significant difference in the distribution of the dihedral angle (p-value = .00374 and .00001, respectively) while R1367 showed no difference (p-value = .4413). **(d)** Comparison of the distances of H1364-E521 and R1367-E521 (H1364 and R1367 from s5/h2 loop and E521 from helix 10) between the main conformation of the WT (blue) and the main conformation of the F583A mutant (red). The right figure presenting the shift between the WT’s residues to the mutant residues, by black arrows.

Differences between the WT and the F583A mutant were located both at the macroscopic level and the microscopic level, specifically in helices 10–11 and NBD2. The missing part in the puzzle is how the mutation propagated from the transmembrane helices to the nucleotide-binding site. To answer this question, we scanned for key residues in the communication between the two parts of the protein. We located two residues in the s5/h2 loop, H1364 and R1367, and one residue in helix 10, E521, that together form the communication path (see methods, energy calculations). In the WT protein, the distance between H1364 to E521 stabilizes on an average value of 2 Å while in the mutant it is shakier around an average value of 6 Å. The distance between R1367 and E521 stabilizes on an average value of 3 Å in the WT and 4 Å in the mutant (Fig. 8A).

Next, we performed an interaction energy calculation to analyze the nature of the NBD2-TMD communication (Fig. 8B). For E521-R1367 a salt bridge bond exists through the entire duration of the WT’s and the F583A mutant’s simulations. However, there is a difference in the strength of the bond, presenting an advantage to the WT over the mutant, with an average value of -17 ± 2 kJ/mol and -12 ± 2 kJ/mol, respectively. For H1364 a bond formed in the WT on an average value of -8 ± 2 kJ/mol while in the F583A mutant no bond is established. The energies reflect the nature of the bonds forming between the residues. In the WT protein, the ionizable side chain of the positively charged H1364 and the guanidinium of the positively charged R1367 from s5/h2 are forming a salt bridge with the anionic carboxylate of the negatively charged E521 from the intracellular section of helix 10. As mentioned above, in the WT protein the average distance of H1364 and R1367 from E521 is 2.5 Å and 3 Å, respectively, suitable for the formation of two salt bridges. However, in the F583A mutant, the distance of H1364 and R1367 from E521 is on average 4 Å, enough for preventing the formation of a stable salt bridge as amino acids in a distance greater than 4 Å do not qualify to form a salt bridge^20^.

Having established the putative allosteric effect of the mutation, we further investigated the dihedrals of the key residues. Dihedrals of H1364, R1367 and E521 were calculated, both for the WT and the mutant protein. H1364 and E521 showed a significant difference in the distribution of the dihedral angle of the WT and the F583A mutant protein (p-value = 0.00374 and 0.00001, respectively). R1367 showed no difference (p-value = 0.4413) (Fig. 8C).

Moreover, a comparison of the most common conformation during the simulation (cluster #1) of the WT and the mutant reveals profound differences. H1364 and R1367 rearrange differently in the protein’s conformational space in the mutant relative to the WT, thus become further away from E521 (Fig. 8D). In The WT, the distances between the groups forming the salt bridge of H1364-E521 and R1367-E521 are 2.4 Å and 3.2 Å, respectively, while in the mutant the distances increase to 5.9 Å and 3.6 Å, respectively, and therefore only one salt bridge is formed. Our findings are supported by an additional methodology, an elastic network model (ENM), presented in a recent study showing that E521 together with H1364 and R1367 are involved in preserving the completeness of the TMD-NBD2 interface and are important for the hMRP1 transport^9^. Considering the differences in the distances and dihedral angles of H1364-E521 and R1367-E521 it is possible to conclude that the optimal terms for the formation of a communication between the transmembrane region and the ATPase region of the protein are not taking place in the mutant protein.

## Discussion

ABC transporters are one of the largest and oldest protein superfamilies found in all phyla taxa, coupling ATP binding and hydrolysis with translocation of a wide array of substrates across the membrane. Although a vast amount of structural and biochemical data was accumulated about their mechanism of action (e.g.,^3,4,8,21^), a few questions are yet to be answered, for example, how does a specific point mutation exert its influence on these transporters? To tackle that question, we extensively studied the effect of a nonconservative substitution F583A, located on the exofacial segment of MRP1, on the activity and structural dynamics of this transporter. In our previous study^8^ , we found that although the F583A mutation did not noticeably affect ATP binding, the ability to release ADP after ATP hydrolysis was markedly decreased. We attributed these findings to either steric constraints imposed on the NBDs or to an increased affinity of the mutant for ADP (> fivefold relative to the WT MRP1). We concluded that the F583A mutation induces changes in the ATP interactions with the transporter, leading to a disruption of the functional cooperativity between the NBDs. This leaves the transporter at a “locked” (trapped) post-hydrolytic, low-affinity substrate-binding state. Thus, rather than merely affecting the substrate selectivity of MRP1, the NBD’s interaction was substantially altered as well. These observations were highly surprising because the exofacial location of F583 at ECL5 of MRP1 is 75 Å far-off from the NBDs.

Since neither the current structures and models of MRP1 nor the biochemical and mutagenesis studies offer substantive mechanistic insights with respect to how the loss of the exofacial F583 alters the nucleotide interactions of MRP1, a further in-depth investigation was needed to understand precisely how the mutation's effect is transmitted across the membrane. It was still to be determined how long-range interactions ranging from a single amino acid span the two bundles of helices (TMD1 and TMD2) of MRP1 and affect the two NBDs. To that end, herein we used modeling and MD simulations, combined with thorough macroscopic and microscopic-scale analyses, and successfully tracked the mechanistic progression of the allosteric mutation from the ECL5 loop, via the helices’ bundles, up to the NBDs. To the best of our knowledge, this is the first MD study that is based on the newly determined bovine MRP1 cryo-EM structure^3^.

**Figure 9:**
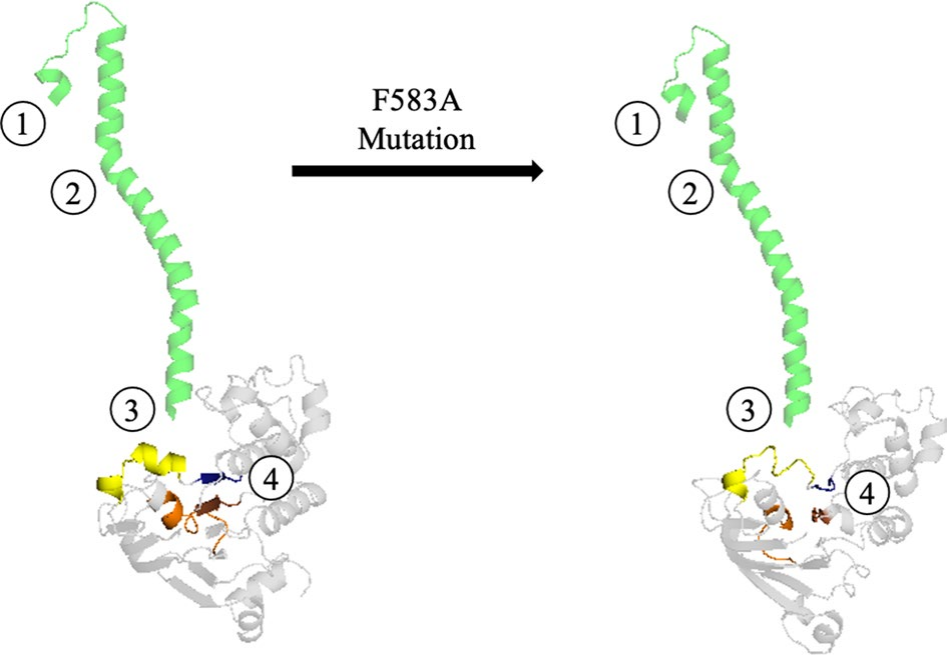
The allosteric pathway. F583A mutation (1) induces a change in helix 10’s angularity, making it more obtuse (2). The shifted bending propagates to the edge of helix 10, deviating its docking orientation in relation to NBD2, which leads to a disruption in the interface communication between the two functional sites of MRP1, TMD-NBD2, by influencing the orientations of residues E521 (helix 10), H1364 and R1367 (NBD2) (3). This is followed by the unraveling of important secondary structure elements in NBD2, which are responsible for ATP binding and hydrolysis (4).

Allostery is an intrinsic characteristic of dynamic macromolecules, representing a phenomenon in which a functional or structural change occurs at a given location while it is generated by a perturbation produced by a distant site. Based on our findings we were able to follow the step-by-step structural pathway of the mutation effect, discovering a hidden allosteric pathway embedded in MRP1, and revealing key cryptic residues. This pathway (Fig. 9) is evoked by the F583A mutation that triggers small conformational changes, that in turn transmit the allosteric effect in a wave-like manner along with structural elements and specific amino acids. The F583A mutation (Fig. 9, circle 1) induces a curvature change in helix 10’s angularity. Since helix 10 is composed of two rod-like elements (speared by W553 and V554) and the angle between them is not rigid, the mutation affects the opening of the angle, making it more obtuse and consequently, the helix becomes straighter (Fig. 9, circle 2). This generates a change to the helix’s orientation in relation to NBD2 (Fig. 9, circle 3). As a result, a disruption in the TMD-NBD2 interface communication is evoked by influencing the orientations of residues E521 (helix 10), H1364, and R1367 (NBD2). Lastly, important secondary structure elements in NBD2 which are responsible for ATP binding and hydrolysis are unraveled (4). This exemplifies how the allosteric signal travels from the ECL5 loop to the NBDs and transmits its effect across the membrane. Having portrayed the entire putative embedded structural allosteric pathway that propagates across the membrane to a distance of ~ 75 Å enabled us to identify the chronological sequence of the molecular events.

Our results regarding the secondary structure unraveling of Walker A and Walker B elements (Fig. 7) support the experimental findings^8^ and raise the possibility that the F583A mutant binds ATP, yet the binding itself is impaired due to structural changes in Walker A and Walker B. The altered binding characteristics increase the affinity of the transporter to ADP and therefore diminish the ability to release it, probably because of steric hindrance imposed by the NBDs. This, in turn, interrupts a proper complete hydrolysis mechanism, specifically the release of ADP and returning to the initial high-affinity binding state. Interestingly, the proposed scenario is supported by a recent study regarding the kinetic cycle of MRP1, using single-molecule FRET and cryo-EM^7^. It was indicated that ATP binding accelerates the IF-to-OF transition of MRP1 and slows down the reverse OF-to-IF transition by stabilizing the OF conformation. Taken together, it is not unlikely that disruption of ADP release due to conformational changes in Walker A and Walker B favors the OF conformation. Thus, F583 appears to be critical for the transition of MRP1 from its low-affinity substrate-binding state to a high-affinity binding state during its transport cycle.

Several key residues that play a significant role by mediating the allosteric propagation were identified. These residues can be classified into two groups based on their positions: E521, W553, and V554 located at helix 10, and H1364 and R1367 located at NBD2. Not surprisingly, most of these residues are known to be involved in MRP1’s function and expression: E521A mutant is characterized by a significant decrease at MRP1 level, altered kinetic interactions with ATP, and shows 50% deficiency of transport activity^22^; W553 has a role in controlling the affinity of LTC4 to MRP1^23^; R1367 is necessary for stable MRP1 expression, H1364A and R1367A mutations resulted in reduced MRP1 levels^24^.

In this study, we harnessed the abilities of computational modeling and MD simulations to investigate a well-studied protein. Notably, MD simulations provide a computational tool to probe membrane proteins on a variety of length scales (tenths of angstroms to nm), ranging from nanoseconds to microseconds and even milliseconds. Although usually protein global motions are generally of the order of microseconds to milliseconds or longer, our results indicate long-range coupled motion between separate protein domains on the nanosecond timescale. A significant conformational protein change in a relatively short timescale is biochemically feasible and physically attainable^25–27^. We were able to explain the molecular structural mechanism of the F583A mutation effect by discovering a long-range conformational transmission across the membrane and an unknown putative allosteric pathway in MRP1.

Our work does not only provide a mechanistic structure–function relationship framework for MRP1 but may contribute valuable insights for the design of specific chemosensitizers and chemotherapeutic agents for MRP1. From a broader perspective, we showed that the effect of point mutations at membrane proteins, and specifically ABC transporters, can be tracked over a long-range of tenths of angstroms by computational means, and identified the allosteric hotspots along the pathway. We hope that our study will promote further works that couple computational mechanistic molecular explanations with biochemical data.

## Materials and methods

### Homology modeling

The template used to model the human MRP1 (UniProt: P33527) was the cryo-EM bovine MRP1 (PDB: 5uj9). The high sequence identity (91%) ensures a high success of the modeling process. The human and the bovine amino acid sequences were aligned using MAFFT^28^. Homology models were generated with Modeller 9.4^29^. The model with the best score was assessed for its quality concerning its energy and stereochemical geometry using Procheck 3.5^30^, ERRAT^31^, and WHATCHECK^32^.

### Systems setup

The model with the best score was inserted into a preequilibrated 1-palmitoyl-2-oleoyl-sn-glycero-3-phosphatidylcholine (POPC) bilayer (adopted and modified from^33^). The insertion was done so that the protein’s rough axis was perpendicular to the membrane surface plane, and all colliding lipids and water molecules, within 2 Å of the protein, were removed. The lipids membrane was scaled to 448 lipids by GROMACS tools to fit the protein size^34^. The simulation box was filled with water using the SPC216 model and the system’s total charge was neutralized by adding Na^+^ and Cl^-^ ions to a final concentration of 0.1 M. Then, the apo human MRP1 model was embedded in a membrane by the g_membed package in GROMACS^35^, removing colliding lipids and water molecules. To construct the F583A mutant the Wizard → Mutagenesis option in PyMOL was used^36^.

### MD details

All MD simulations were performed using GROMACS 5.1.4 package (http://www.gromacs.org/)^34^ with AMBER99sb-ildn force field^37^. A total of six systems were created, three for WT apo-protein and three for mutant F583A apo-protein. The systems were subjected to rigorous energy minimization using the steepest descent algorithm with a sequential decreasing tolerance, from 1000 to 200 kJ‧mol^−1^‧nm^−1^. Then, the systems were equilibrated under NPT for 500 ps for position restraint, allowing the solvent to equilibrate around the protein without disturbing the protein structure. Each system was simulated further for 10 ns of unconstrained equilibrium simulation, using Berendsen’s coupling algorithm^38^ for keeping the pressure constant. Afterward, the systems were submitted for unbiased MD production runs of 100 ns each. All simulations were conducted using the LINCS algorithm^39^ to constrain bond lengths and angles of hydrogen atoms, allowing a time step of 2 fs. The pressure was kept constant using the Parrinello-Rahman coupling algorithm at 1 bar by applying a semi-isotropic coupling constant of τ_P_ = 1 ps. Each simulation had a different initial velocity for every atom that was randomly generated from the Maxwell–Boltzmann distribution at 310 K employing a coupling constant of τ_T_ = 0.1 ps. A cutoff of 1.2 nm was used for van der Waals interactions, and long-range electrostatic interactions were computed using the PME method^40^.

### Visualization and analyses

The analyses were conducted using in-house purpose-written python scripts and the GROMACS analysis package utilities^34^. Three WT trajectories and three F583A mutant trajectories were used for our analyses, to ensure statistical significance. All the systems obtained the equilibrated state after 20 ns of the production runs; hence the last 80 ns stable sections of the trajectories were considered for the in-depth analyses. In all the simulations the protein was stable (backbone atom’s RMSD of up to 6.1 ± 0.3 Å for the WT and 6.3 ± 0.6 Å for the F583A mutant; Fig. S1). All snapshots were prepared using PyMOL^36^.

#### Cluster analysis

A geometric clustering algorithm was performed to identify similar structures, based on the respective RMSD between all conformations sampled during the MD simulation^41^. A 3 Å predefined RMSD cut-off was chosen to determine the cluster group, to generate a sufficient number of clusters while maintaining statistical significance, e.g.,^42,43^. The cluster number for each frame was calculated, summarized to a table consisting of the number of frames for each cluster. The average of the three WT simulations and the F583A mutant’s simulations were calculated separately. Then, the corresponding conformation was determined.

#### Evolutionary conservation score

Conservation analysis was performed using the Consurf server^44^ and multiple sequence alignment by ClustalW ^45^.

#### Principal component analysis (PCA)

The covariance matrix was calculated, diagonalized, and analyzed by GROMACS utility tools. The corresponding eigenvalues are plotted against the first five corresponding eigenvector indices obtained from the Cα covariance matrix constructed from the MD trajectory. Visualization projection of the first eigenvector’s obtained results is shown by arrows representing the principal components of the protein’s motion throughout the simulations, from the starting structure to the final structure, using a PyMOL-designed script named Modevectros.py. When all vectors are introduced, there is a wide plethora of visual data that makes it difficult to differentiate between the different arrows and masks the significant movements (Fig. S2). To overcome the data overload, we filtered the significant movements by choosing a cut-off of 4 Å (https://pymolwiki.org/index.php/Modevectors, https://github.com/Pymol-Scripts/Pymol-script-repo/blob/master/modevectors.py).

#### RMSD matrix

RMSD matrix was produced in two-dimensional format to compare values of the WT structure in the trajectory with respect to the F583A mutant structure.

#### Interhelical opening angle between helices 10 and 11

Interhelical angles were calculated according to the following procedure: One residue was selected at the C-terminal of helix 10 (I571), one residue at the N-terminal of helix 11 (I577), and one residue at the ECL5 loop (N574). The Cα atoms’ center of mass of the residues was determined. A vector-defining helix was drawn between these centers of mass along each helix. The interhelical angle was calculated between every two successive vectors.

#### DSSP (define secondary structure of proteins) algorithm

Secondary structure content was determined with the STRIDE algorithm^46^.

#### Energy calculations

To discover key residues in the communication between helix 10 and NBD2 we filtered residues in the upper side of NBD2 that are located within an average distance shorter than 3 Å from the lower part of helix 10, both in the WT and the F583A mutant. Energy calculations (vdW + Electrostatic) were done using the MM-PBSA method for GROMACS for each of the filtered residues^47^. Only two residues presented energy differences between the WT protein and the F583A mutant, H1364 and R1367. We chose to use a dielectric constant of ε = 20 because the residues we analyzed are located on the protein’s surface and electrically charged^48^.

#### Dihedral angles

Atoms defined for dihedral calculations were: Cα-Cβ-Cγ-Cδ2 for H1364, Cδ-Nε-Cζ-Nη2 for R1367 and Cβ-Cγ-Cδ-Oε1 for E521.

## Supplementary Information

Supplementary Figures.
