# Supplementary material for: Extracellular mutation induces an allosteric effect across the membrane and hampers the activity of MRP1 (ABCC1)
Source: Sci Rep. 2021 Jun 8;11:12024. doi: 10.1038/s41598-021-91461-3 (PMC8187718; doi:10.1038/s41598-021-91461-3)
Supplement: Supplementary file 1 — Supplementary Figures. [file 41598_2021_91461_MOESM1_ESM.pdf]

Extracellular Mutation Induces an Allosteric Effect across the Membrane and Hampers the Activity of MRP1 (ABCC1)

Yuval Bin Kanner [a], Assaf Ganoth [b,c] and Yossi Tsfadia [a*]

[a] The School of Neurobiology, Biochemistry and Biophysics, George S. Wise Faculty of Life Sciences, Tel Aviv University, Tel Aviv 69978, Israel.

[b] The Interdisciplinary Center (IDC), P.O. Box 167, Herzliya 4610101, Israel.

[c] Department of Physical Therapy, School of Health Professions, Sackler Faculty of Medicine, Tel Aviv University, Tel Aviv 69978, Israel.

[*] Corresponding author.

Supplementary Information

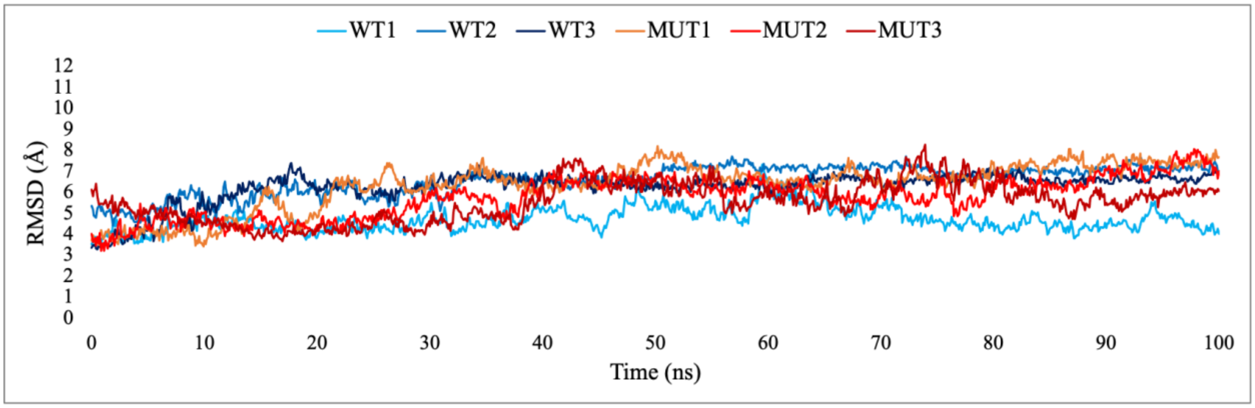

Fig. S1. Backbone atoms' RMSD analysis as a function of the simulation time for the WT (blue colors) and the F583A mutant (red colors). For visual clarity, each simulation system is depicted by a different color as indicated in the inset.

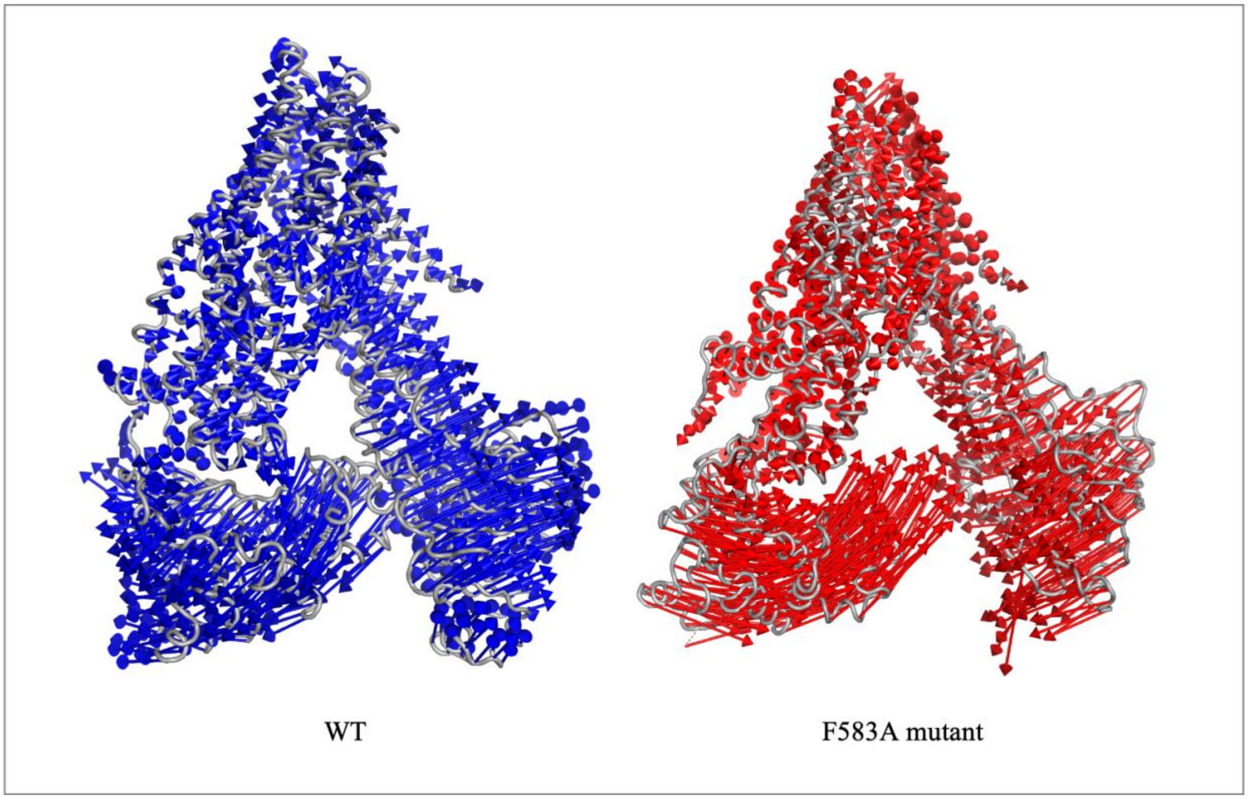

Fig. S2. Principal Component Analysis (PCA) of WT (blue) and F583A mutant (red) MRP1. Visualization projection of the raw data of the first eigenvector. The principal components of the protein's motion throughout the simulations are represented by arrows. The lengths of the arrows are proportional to the size of the movement. Figure was created using Modevectros.py, a PyMOL-designed script.
